# New and High Virulent Pathotypes of Sunflower Downy Mildew (*Plasmopara halstedii*) in Seven Countries in Europe

**DOI:** 10.3390/jof7070549

**Published:** 2021-07-10

**Authors:** Rita Bán, Attila Kovács, Nisha Nisha, Zoltán Pálinkás, Mihály Zalai, Ahmed Ibrahim Alrashid Yousif, Katalin Körösi

**Affiliations:** 1 Department of Integrated Plant Protection, Plant Protection Institute, Hungarian University of Agriculture and Life Sciences, H-2103 Gödöllő, Hungary; nisha27evs@gmail.com (N.N.); Palinkas.Zoltan@uni-mate.hu (Z.P.); Zalai.Mihaly@uni-mate.hu (M.Z.); ahmadalrashed45@gmail.com (A.I.A.Y.); Korosi.Katalin.Orsolya@uni-mate.hu (K.K.); 2 Syngenta Kft., H-1117 Budapest, Hungary; Attila.Kovacs@syngenta.com

**Keywords:** pathotype distribution, downy mildew of sunflower, pathotype characterization, coded virulence formula (CVF)

## Abstract

Downy mildew of sunflower, caused by *Plasmopara halstedii* (Farl.) Berl. et de Toni, is a relevant disease of this crop. High virulent pathotypes have been identified in several countries, while there are few data on the spread of *P. halstedii* pathotypes in some important sunflower-growing areas of Europe. The goal of this study was to give up-to-date information on the pathotype structure of *P. halstedii* in Hungary and provide some actual data on the virulence phenotype of the pathogen for six European countries. Infected leaves of different sunflower hybrids and volunteers were collected in seven countries (Hungary, Bulgaria, Serbia, Turkey, Greece, Romania, and Italy) between 2012 and 2019. A universally accepted nomenclature was used with a standardized set of sunflower differential lines for pathotype characterization of isolates. The virulence pattern of the isolates was determined by a three-digit code (coded virulence formula, CVF). A total of 109 *P. halstedii* isolates were characterized. As a result of our survey, 18 new *P. halstedii* pathotypes were identified in Europe. Two out of the eighteen pathotypes were detected from the Asian part of Turkey. The detailed distribution of pathotypes in Hungary is also discussed.

## 1. Introduction

Sunflower (*Helianthus annuus* L.) is among the world’s most important oilseeds. As sunflower seed oil is one of the healthiest vegetable oils available for cooking, there is increasing demand due to a health-conscious diet [1]. The cultivation of sunflower is significantly affected by diseases. *Plasmopara halstedii* (Farl.) Berl. et de Toni, a biotrophic oomycete pathogen, is the causal agent of downy mildew in sunflower. The pathogen has been reported as an essential factor affecting yield in sunflower-producing countries. Besides the control methods currently deployed against the disease, yield reduction can be relevant, representing an average of 3.5% of commercial seed production. Heavy infections can cause up to 100% yield loss and make sunflower growing impossible in those fields [2].

*Plasmopara halstedii* causes various symptoms depending mainly on the host stage at infection [3]. Primary infection followed by direct movement of zoospores toward the roots causes dwarfing of diseased plants, chlorosis along the veins of the leaves and small heads with sterile seeds [4]. Heavy infection can result in damping off. Secondary infections by zoospores and sporangia that develop underside the leaves are neither significant for spreading the disease nor crop loss. Besides local symptoms, however, secondary infections sometimes turn systemic, causing dwarfism of affected plant parts [5]. Additionally, secondary infection poses a risk that the disease can spread latently with the seeds.

Seed treatment with fungicides provides an efficient way to protect young sunflower plants against primary infection by zoospores from the soil. There are only a few active ingredients such as mefenoxam that are effective enough against *P. halstedii.* The widespread and exclusive use of this chemical resulted in a descending efficiency and has led to the development of resistance or tolerance in the pathogen worldwide [6,7,8].

*Plasmopara halstedii* has several pathotypes (or races) with different degrees of virulence. The reason for this high variability is the widespread growing of sunflower hybrids with an increasing number of developed resistance genes against *P. halstedii* that induce changes in the genome of the pathogen [2]. Besides mutation and sexual recombination, another main driving force is parasexual recombination providing an opportunity for genetic exchange between different pathotypes [9,10].

The number of pathotypes is continuously increasing worldwide and even accelerated in the last decade. Gulya [11] has previously reported on 35 pathotypes. More recently, new global and highly aggressive pathotypes of *P. halstedii* have been identified in some areas of Europe, such as pathotype 354 in Germany [12], 724 and 734 in Hungary [13,14], 705 in Spain [15], and 705 and 715 in the Czech Republic [16]. Altogether, there are as many as 50 pathotypes worldwide [17,18]. Furthermore, different pathotypes spread into new areas, making sunflower hybrids with earlier resistance genes challenging to grow [19,20]. The scarcity of data on the spread of *P. halstedii* pathotypes in some important sunflower-growing areas of Europe (e.g., in the Balkan countries) also complicates the situation [21].

Dominant resistance genes incorporated into sunflower hybrids give protection against downy mildew only for a limited time because of the high variability of the pathogen [7]. Hence, testing the pathotype composition (virulence character) of *P. halstedii* populations plays a vital role in the breeding activity. A significant outbreak of downy mildew caused by a new *P. halstedii* pathotype (704) was reported from two commercial sunflower fields in Hungary in 2011 [22]. Obtaining new information on the pathotype composition of sunflower downy mildew became urgent; therefore, the primary goal of this study was to give up-to-date information on the pathotype structure of *P. halstedii* in Hungary. Meanwhile, we also received infected sunflower samples from other countries, such as Bulgaria, Serbia, Turkey, Greece, Romania, and Italy, with severe outbreaks of downy mildew. Hence, another objective of this study was to provide some actual data on the virulence phenotype of the pathogen for these countries without attempting a detailed situation analysis.

## 2. Materials and Methods

### 2.1. Collection of Downy Mildew-Infected Sunflowers

Infected leaves of different sunflower hybrids, all carrying the *Pl*6 resistance gene against *P. halstedii*, were collected in 7 countries between 2012 and 2019 (Table 1). In some cases, the samples were collected from volunteer sunflower plants between rows. In our study, samples from the same leaf of the same plant are called isolates. A total of 109 *P. halstedii* isolates were characterized. Field samples of the pathogen originated from central and southern Europe (Hungary, Bulgaria, Serbia, Thracian region in Turkey, Greece, Romania, and Italy) and a small part from the Asian region (Salbas: Adana region) of Turkey. Sunflower fields in Hungary were screened regularly for downy mildew in central sunflower-producing areas during the examination period, but the collection of samples was limited to years when higher levels (approximately 5–50%) of disease occurred. Other (not Hungarian) isolates mostly came from samples collected in fields where heavy downy mildew infection was detected. After sampling, the isolates were frozen to −70 °C as soon as possible and stored until use.

### 2.2. Increase of Inoculum

The inoculum was increased in a susceptible Hungarian sunflower cultivar (cv. Iregi szürke csíkos), which does not possess any resistance genes to the pathogen. Untreated seeds of cv. Iregi szürke csíkos were obtained from Iregszemcse Research Station of Hungarian University of Agriculture and Life Sciences (Hungary). Preparation of inoculum was performed according to Trojanová et al. [23]. Sunflower seeds were surface sterilized with 1% NaOCl for 3 min, then rinsed in distilled water and incubated between wet filter paper for three days at 19 °C until radicles reached a length of 2 to 5 cm.

The whole seedling immersion (WSI) method was used for inoculation [24]. Sunflower leaves frozen at −70 °C, showing intense sporulation, were placed in bidistilled water, and the sporangia, which form a white coating, were washed off the surface with a brush. The inoculum concentration was measured by the Burker chamber and adjusted to 5 × 10^4^ sporangia per mL. Seedlings were then immersed in the sporangial suspension at 16 °C for 5 to 6 h or overnight.

Inoculated sunflower seedlings were sown in horticultural perlite (d = 4 mm, Hungarian Perlit Ltd.) and kept in a growth chamber (22 °C, 12 h photoperiod, light irradiance of 100 μE·m^−2^·s^−1^) for 8 to 10 days. After the first pair of true leaves appeared, sunflowers were incubated for 24 h at 100% relative humidity at 19 °C in the dark for sporulation. Collected sporangia were used as the inoculum for subsequent pathotype characterization.

### 2.3. Pathotype Characterization

**Figure 1:**
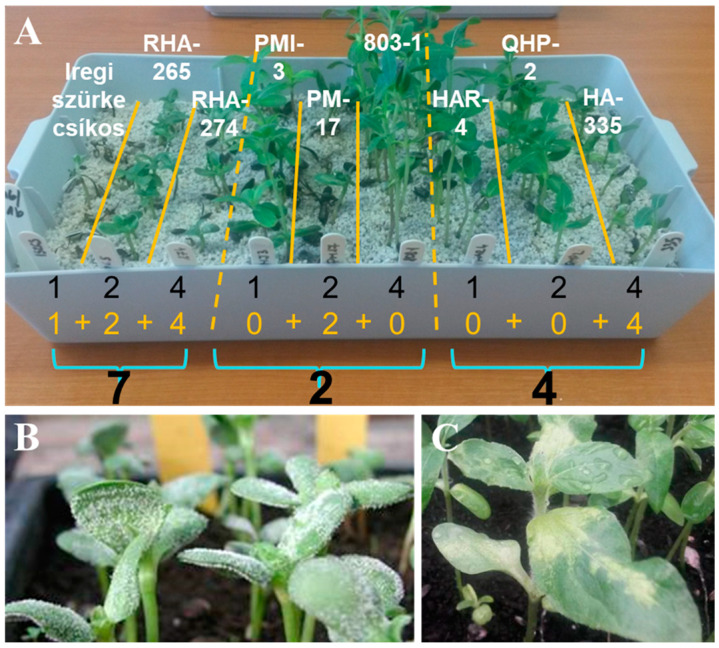
The pathotyping process of *Plasmopara halstedii* (photo: R. Bán). (**A**) The differential sunflower set. Except for the first line, each possesses specific resistance genes against *P. halstedii*. Differential lines are grouped into three sets of three lines. If the sunflower line is susceptible, a value of 1, 2, and 4 is assigned to the first, second, and third lines within each group, respectively. In the case of resistant reaction, the value is 0. The values are added within each group, which results in a three-digit code (coded virulence formula, CVF). The identification of pathotype 724 is shown as an example. (**B**) Signs of *P. halstedii* on the cotyledons. The disease was first assessed based on the formation of white sporulation. (**C**) Symptoms of *P. halstedii* on the true leaves. A second evaluation was made 21 days after inoculation based on the appearance of white coating and chlorotic lesions on true leaves and stunting of plants. Differential lines were assessed as susceptible if sporulation occurred on the cotyledons and/or chlorosis appeared along the veins of the true leaves.

For pathotype characterization of *P. halstedii* isolates, a universally accepted nomenclature was used with a standardized set of sunflower differential lines [23,25]. The differential sunflower set includes nine differential lines (Table 2). Except for the first line, each possesses specific resistance genes against *P. halstedii* (in many cases, we used Iregi szürke csíkos, an open-pollinated Hungarian sunflower cultivar as a susceptible line, instead of HA-304). Differential lines are grouped into three sets of three lines (Figure 1A). If the sunflower line is susceptible, a value of 1, 2, and 4 is assigned to the first, second, and third lines within each group, respectively. In the case of a resistant reaction, the value is 0. The values are added within each group, which results in a three-digit code (coded virulence formula, CVF). The CVF provides information about the virulence pattern of the isolate.

Germination, inoculation, and growth of the seedlings were performed as described above. After 8 to 10 days, sporulation was stimulated by spraying bidistilled water onto sunflower plants and covering them with dark bags for eight hours. The disease was first assessed based on the formation of white sporulation on cotyledons (Figure 1B). A second evaluation was made 21 days after inoculation based on the appearance of white coating and chlorotic lesions on true leaves and stunting of plants (Figure 1C). Differential lines were assessed as susceptible to sporulation on the cotyledons and chlorosis along the true leaves’ veins. Experiments were conducted twice with two replicates each.

## 3. Results

### 3.1. Result of Pathotype Characterization

The results of the pathotype characterization of isolates collected from different years and areas are shown in Table 3. Out of the 70 Hungarian isolates, 26 have already been characterized and published earlier (see Discussion). Therefore, new records are highlighted with bold isolate numbers in Table 3. However, it was considered appropriate to present published and new results from 2012 to 2019 together.

An unexpected result was that we identified less virulent pathotypes (such as 700, 710, and 730) from hybrids with the *Pl*6 resistance gene conferring resistance to these pathotypes (Isolates 16, 22–23, 35–36, 46, 81, 90, 93, and 101). In five cases, we also collected samples from volunteer plants. On two of these plants, low and high virulent pathotypes with 0 and 4 in digit 3, respectively, were found together (Isolates 3–6 and 78–79). On three volunteer plants, low virulent pathotypes (Isolates 34 and 62) and high virulent ones (Isolate 63) were identified. Moreover, in several cases, isolates collected from the same plant but different leaves could be classified into different pathotypes, such as Isolates 3–6, 18–19, 26–29, and 39–42 (Table 3).

**Figure 2:**
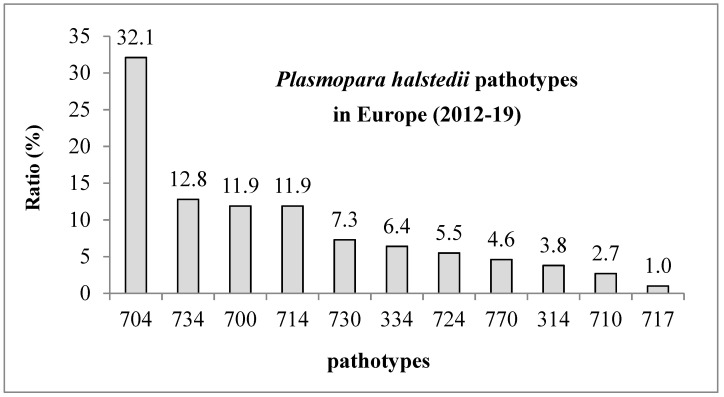
The overall distribution of *Plasmopara halstedii* pathotypes in 7 countries in Europe (Hungary, Bulgaria, Serbia, Turkey-Thracian, Greece, Romania, and Italy) and Asian region of Turkey (2012–2019). The ratio of pathotypes is based on examining 109 isolates: 70 isolates from Hungary, 10 isolates from Bulgaria, 2 isolates from Serbia, 8 isolates from Turkey, 3 isolates from Greece, 13 isolates from Romania, and 3 isolates from Italy.

The 109 isolates could be classified into 11 different pathotypes (Figure 2). Looking at the pathotype distribution in seven different countries together, pathotype 704 was the most common in the collected samples, but pathotypes 734, 700, and 714 were also distributed between 2012 and 2019.

### 3.2. Regional Occurrence of P. halstedii Pathotypes

The distribution of different *P. halstedii* pathotypes in seven countries between 2012 and 2019 is represented in Table 4. Outside Hungary, the most pathotypes were detected in Romania (seven), Bulgaria (five), and Turkey (four), and only two were identified in Greece, Italy, and Serbia, respectively. In Hungary, pathotype 704 was the most widespread, while pathotypes 700, 714, and 730 were also identified. Pathotype 724, identified in 2017, and 734, detected in 2019, were less frequent together with 710. It is remarkable that between 2012 and 2014, 36% of the Hungarian isolates tested belonged to the low virulent pathotypes (700, 710, 730) identified before 2010 and 64% to the high virulent ones (704, 714) (Table 3). In 2016–2019, the former rate slightly changed in Hungary, with almost 80% of isolates being pathotypes with higher virulence (704, 714, 724, 734).

Similar pathotypes of sunflower downy mildew were identified in Romania and Bulgaria over the period. Pathotypes 314, 730, and 734 were common in the collected samples in both countries, while 704 and 770 were more widespread in Romania (Table 4). Pathotype 724 only occurred in Romania and Hungary. Four pathotypes, 314, 334, 734, and 770 of *P. halstedii*, are new records in both countries. In addition, we are the first to publish two further pathotypes, 704 and 724, from Romania (Table 3).

In Turkey, pathotypes 704 and 714 were identified in the Asian part (Salbas: Adana region), while pathotypes 334 and 734 were isolated in the European part (Thracian region) in 2015 (Table 3). Pathotype 334 was dominant in the samples collected in this country. These four pathotypes are the first to be identified from Turkey.

During our survey, pathotype 717 could only be identified from Italy, and pathotype 700 from Serbia (outside Hungary) (Table 4), but only a few samples from these countries and Greece have reached our laboratory. Pathotype 717 and 704, respectively, are newly detected from Italy and Serbia. Out of the three Greek isolates, two were characterized as pathotype 734 and one as 704. Both are new records in this country.

## 4. Discussion

Knowledge of the distribution of *P. halstedii* pathotypes is of utmost importance for effective pest management; however, there is only limited information about pathotype diversity for some vital sunflower-growing countries [18,21,26].

Over the past nine years, we have received and collected samples from Hungary in different regions and other countries where severe outbreaks of sunflower downy mildew have occurred. In this paper, we reported 18 new pathotypes in six countries, where very few or no data on the occurrence of *P. halstedii* pathotypes were available before. New pathotypes were detected in Bulgaria (314, 334, 734, 770), Serbia (704), Turkey (334, 704, 714, 734), Greece (704, 734), Romania (314, 334, 704, 724, 734, 770), and Italy (717). Out of the 18 new records, two (704 and 714) originated from the Asian part of Turkey.

Updated information on the distribution and ratio of different *P. halstedii* pathotypes in Hungary is also a new survey result. Before 2010, pathotypes 100, 330, 700, 710, and 730 were considered relevant in Hungary [11]. In a recent paper, Viranyi et al. [21] pointed out that there was a significant shift in the virulence character of *P. halstedii* populations detected between 2007 and 2013 either in Hungary or worldwide. The first high virulent pathotype (704) was isolated in 2010 by Rudolf et al. [22] from two sunflower fields in Hungary. In the following years, our research team confirmed the increased distribution of pathotype 704 in the country [27] and the emergence of three new *P. halstedii* pathotypes, 714, 724, and 734 [13,14,28] (New records are separated from previously published records by bold isolate numbers in Table 3.). Previously, pathotype 724 was only identified from Hungary, but according to our present results, it was also detected in Romanian samples in 2019.

Viranyi et al. [21] underlined that, despite new pathotypes, pathotypes 700 and 730 were still predominant in Hungary from 2007 to 2014. According to our results, based on the characterization of 42 isolates from the central sunflower-producing regions in Hungary, the dominant distribution (64%) of high virulent pathotypes was proven between 2012 and 2014. These strains could overcome the protective effect of the *Pl*6 resistance gene incorporated into a wide range of sunflower hybrids. Among the less virulent ones, pathotype 700 continued to be dominant, while pathotypes 100 and 330 seem to have disappeared. In addition, Körösi et al. [8] pointed out that pathotype 704 was rather widespread in Hungary between 2014 and 2017. From our findings, a further shift in the composition of the pathotypes could be detected from 2016, with an almost 80% occurrence of more virulent pathotypes. To fully prove this pathogenic shift, however, many more samples and more frequent sampling would be needed.

It seems likely that the spreading of new, high virulent pathotypes has accelerated since 2012 in Hungary. The reason for the appearance and spreading of new *P. halstedii* pathotypes could be the result of several factors, including mainly the presumed high allele frequency of either the *Pl*6, the more and more advanced and newest tolerance genes in the new sunflower hybrids supplied by international breeding and seed companies, and the more frequent favorable weather conditions for *P. halstedii* in the concerned period. In addition to the above, the appearance of mefenoxam-tolerant *P. halstedii* pathotypes [8], short crop rotations, and the spread of minimum tillage systems may accelerate the emergence of increasingly aggressive pathotypes.

The samples collected outside Hungary are not representative of the pathotype pattern in the country, but several new data have been revealed. Out of the ten isolates collected in Bulgaria, four new pathotypes (314, 334, 734, 770) were identified. Previously, Shindrova [29,30] and Spring [18] reported the distribution of *P. halstedii* pathotypes in Bulgaria, but from these, only pathotype 730 could be detected in our survey. Similarly, of the previously described pathotypes in Romania [18], only 730 was identified in our study, and the majority of the isolates (six) could be described as new pathotypes to the country. These are 314, 334, 704, 724, 734, and 770. Recently, Miranda-Fuentes et al. [26] has identified pathotype 705 from Romania.

Similar to Spring [18], concerning the pathogenic diversity of *P. halstedii* in Serbia, the occurrence of pathotype 700 was proven in our study. In addition, the appearance of pathotype 704 is a new record from this country. Besides pathotype 734, 704 was also found in Turkey, but pathotypes 334 and 714 were predominant in the collected samples. None of these is mentioned in earlier studies [18,31]. Of the four newly identified pathotypes in Turkey, two (704, 714) originated from the Asian part, where only a few data about the virulence character of *P. halstedii* exist. Some results are available from China and India (pathotypes 100 and 300 in both countries) as well as Iran with only pathotype 100 [18]. Although these are new data from the Asian region, they represent a total of three samples, which does not allow us to draw any significant conclusions about the population composition of *P. halstedii* in Asian regions. Similarly, there are no distribution data for sunflower downy mildew in Greece, where two high virulent pathotypes (704 and 734) were detected during our survey.

Earlier in Italy, Tosi and Zazzerini [32] and Tosi and Beccari [33], as well as Spring [18], published results about the spread of virulent pathotypes. More recently, Miranda-Fuentes et al. [26] confirmed pathotypes 301 and 715 in Italian samples. We identified pathotype 717 for the first time in Italy. In addition, Martin-Sanz et al. [34] reported on a record of pathotype 714, which could overcome even the *Pl*8 resistance gene. With this, the latter authors highlighted the bottlenecks of current pathotyping methods.

As the variability is increasing in the *P. halstedii* population worldwide, the currently used pathotyping system no longer provides sufficient information on the virulence character of this pathogen, indicated by many authors [18,21,23,34]. The inclusion of new lines with advanced resistance genes in the differential set was proposed by Gascuel et al. [2], but the complementary set is not widespread. One of the main advantages of the new set is that it fits well with the previous one so that earlier results are still prevailing. We plan to test the isolates in our collection on the new differential set to give more exact information on their CVF in the future. However, as researchers widely accept it, only molecular methods are likely to provide a long-term and reliable solution in more precise pathotyping [18].

Currently, a significant proportion of sunflower hybrids contain advanced resistance genes against *P. halstedii*, an essential agronomic trait in integrated plant protection. However, to a smaller extent, less virulent pathotypes such as 700, 710, and 730 were also present in the pathogen populations and could be identified from sunflowers with resistance genes against these strains. Moreover, less virulent and high virulent pathotypes could be isolated from the same plant in several cases simultaneously. The exact reason for this is still unknown, but similar cases are discussed by other authors. Studying wheat–*Zymoseptoria tritici* interactions, Kema et al. [35] demonstrated that during coinfection by two different fungal strains, an avirulent strain could fertilize the female organ of the virulent strain upon penetration, thus allowing transmission of avirulence genes to the progeny. Moreover, Seybold et al.’s [36] findings suggest that immune suppression of wheat by virulent strains of *Z. tritici* predisposes the plants to further infections by induced susceptibility. Accordingly, a highly aggressive pathotype likely represses the host’s defense mechanisms, creating favorable conditions for the less virulent (or avirulent) pathotypes. It is even likely that lower virulence in these strains is associated with higher fitness, contributing to their persistence [37]. The background of this phenomenon still has to be elucidated.

Integrated plant protection is an essential tool to manage pests in sunflower production. Incorporating resistance genes and combining qualitative and quantitative resistance by maximizing the diversity of genes in sunflower hybrids against *P. halstedii* is a continuous work in sunflower breeding [38,39]. It is vital that the *Pl*6 resistance gene is ineffective in most countries due to the emergence of new, more virulent pathotypes, as indicated in our study. Weed management also plays an important role in disease control because many weeds are host plants for the pathogen [7,40]. Eradication of volunteer plants is also essential; hence, they can serve as reservoirs for less and high virulent pathogen variants, also indicated by our results. Finally, the application of new active substances, such as oxathiapiprolin, is fundamental to achieve adequate chemical protection against *P. halstedii* [41,42].

## 5. Conclusions

Based on the pathotype characterization of 109 *P. halstedii* isolates, it can be assumed that there is a shift in the virulence character of *P. halstedii* towards more virulent pathotypes. Furthermore, the occurrence of low virulent pathotypes, such as 700, 710, and 730, was proven in sunflowers with resistance genes against these variants. Induced susceptibility, one possible reason for this phenomenon, is also discussed by other authors [35,36]; it seems likely that this, together with higher fitness, can result in the long viability of these “old” pathotypes in the population.

The currently used pathotyping system has many advantages and several weaknesses [23]. The addition of other sunflower differential lines to the system and their widespread use will provide a temporary solution, and the results of the previous identifications will not be lost [2,18]. Thus, earlier records will be compatible and more accurate with the new, enhanced system, but the inclusion of molecular tests in this process cannot be delayed much longer. In addition, the broader use of integrated plant protection could significantly slow down the evolution of new pathotypes, not only for *P. halstedii* but also for other plant pathogens.

## Figures and Tables

**Table 1:** List of *Plasmopara halstedii* isolates collected from seven countries in Europe (2012–2019).

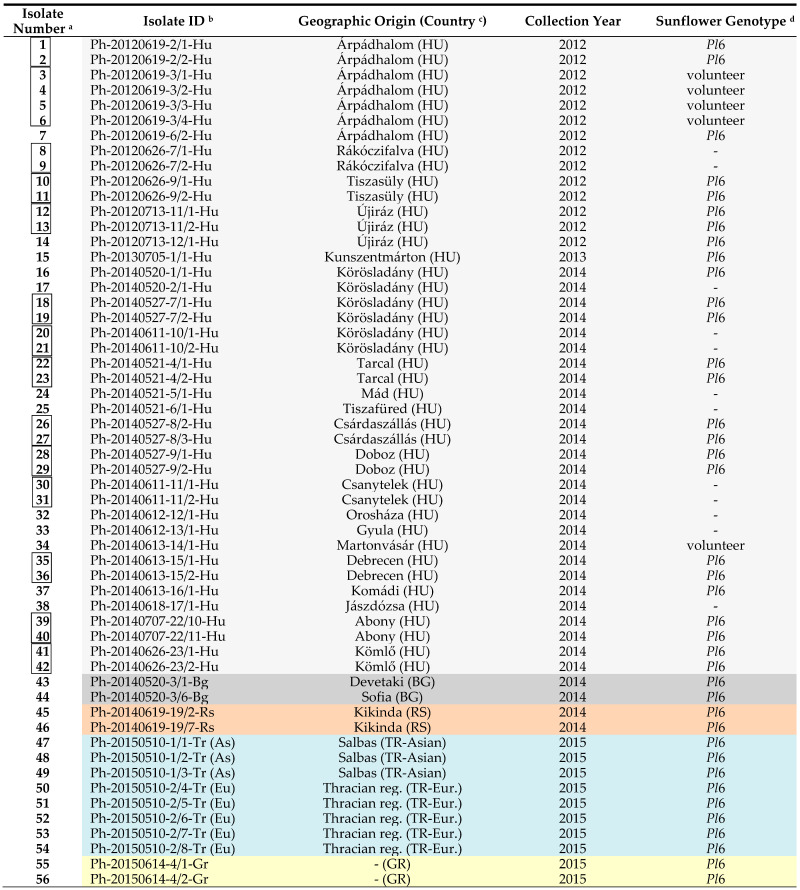
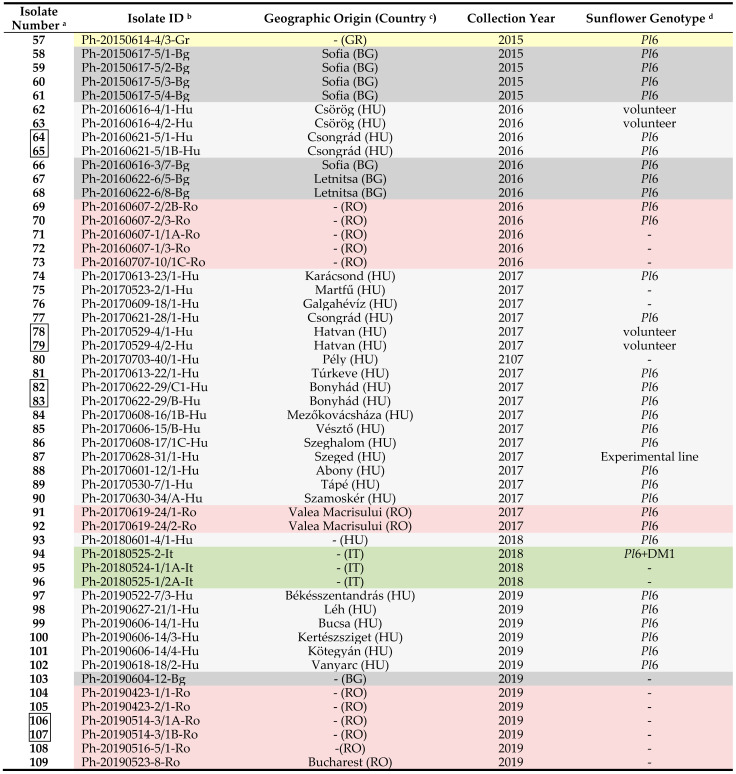

^a^ linked isolates are from different leaves of the same plant. ^b^ the isolate ID contains the collection date (year-month-day), the code in MATE collection (Gödöllő, Hungary), and country. ^c^ HU: Hungary, BG: Bulgaria, RS: Serbia, TR-Asian: Turkey (Asian part), TR-Eur.: Turkey (European part), GR: Greece, RO: Romania, IT: Italy. ^d^ unknown, *Pl*6: hybrid with *Pl*6 resistance gene to *P. halstedii*.

**Table 2:** Sunflower differential lines with different resistance genes used in the experiment for pathotype characterization of *Plasmopara halstedii* (based on Gascuel et al. [2], Trojanová et al. [23], and Gulya et al. [25]).

Sunflower Differential Line	HA-304 ^a^	RHA-265	RHA-274	PMI-3	PM-17	803-1	HAR-4	QHP-2	HA-335
Resistance gene to *P. halstedii*	No *Pl* gene	*Pl*1	*Pl*2*/Pl*21	*Pl_PMI3_*	*Pl*5	*Pl*5*+*^b^	*Pl_15_*	*Pl*1*/Pl_15_*	*Pl*6

^a^ alternatively, in several cases, Iregi szürke csíkos, an open-pollinated Hungarian sunflower cultivar, was used; ^b^ *Pl*5*+* stands for a stronger allele of *Pl*5.

**Table 3:** Virulence character of *Plasmopara halstedii* isolates collected from seven countries in Europe (2012–2019).

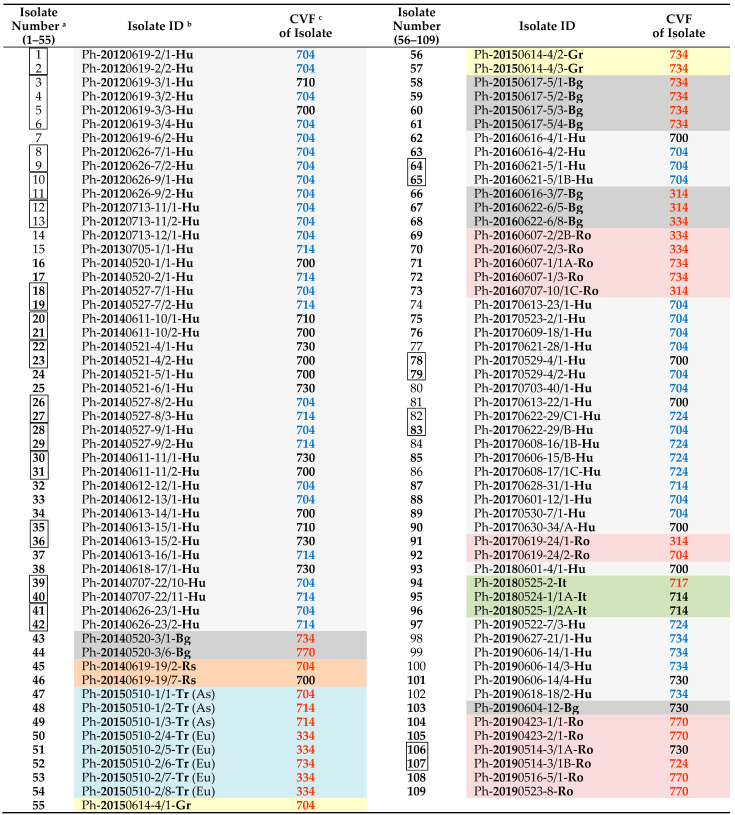

^a^ linked isolates are from different leaves of the same plant. ^b^ the isolate ID contains the collection date (year-month-day), the code in MATE collection (Gödöllő, Hungary), and the country: Hu: Hungary, Bg: Bulgaria, Rs: Serbia, Tr: Turkey (As: Asian part, Eu: European part), Gr: Greece, Ro: Romania, It: Italy. ^c^ coded virulence formula. New pathotypes to a region are highlighted in orange. Pathotypes in blue have been identified in Hungary since 2010. CVFs for isolates with bold isolate number have not been published before.

**Table 4:** Distribution of *Plasmopara halstedii* pathotypes by countries in Europe (2012–2019).

				Ratio of Pathotypes (%)							
Country	704	734	700	714	730	334	724	770	314	710	717
Hungary	44.3	5.7	17.1	12.9	8.6	0	7.1	0	0	4.3	0
Bulgaria	0	50	0	0	10	10	0	10	20	0	0
Serbia	50	0	50	0	0	0	0	0	0	0	0
Turkey	12.5	12.5	0	25	0	50	0	0	0	0	0
Greece	33.3	66.7	0	0	0	0	0	0	0	0	0
Romania	7.7	15.4	0	0	7.7	15.4	7.7	30.7	15.4	0	0
Italy	0	0	0	66.7	0	0	0	0	0	0	33.3

## Data Availability

Not applicable.
